# Endothelin-1: a multifunctional molecule in cancer

**DOI:** 10.1038/sj.bjc.6700750

**Published:** 2003-01-28

**Authors:** K Grant, M Loizidou, I Taylor

**Affiliations:** 1 Department of Surgery, Royal Free and University College London Medical School, University College London, UK

**Keywords:** endothelin-1, cancer, angiogenesis, tumour growth, apoptosis

## Abstract

Endothelin-1 is a small vasoconstrictor peptide that was first identified in 1988. Here we review the evidence implicating ET-1 in tumorigenesis. In particular, we concentrate on the role of ET-1 in mitogenesis, apoptosis, angiogenesis, tumour invasion and metastasis, and discuss the potential for endothelin-system modulation as an adjuvant therapeutic strategy.

The potent vasoconstrictor peptide endothelin-1 (ET-1) was first isolated from the culture media of porcine endothelial cells in 1988 (Yanagisawa *et al*, 1988). It is one of a family of multifunctional peptides (ET-1, 2 and 3) that are closely related to the sarafotoxins derived from the venom of the burrowing asp. Of these isoforms, ET-1 has been the most extensively studied to date, and has been implicated in cancer.

ET-1 is synthesised via proteolytic cleavage of a large precursor molecule, pre-pro ET-1, which is facilitated by the metalloproteinase, endothelin converting enzyme (ECE). This pathway is summarised in Figure 1

**Figure 1:**
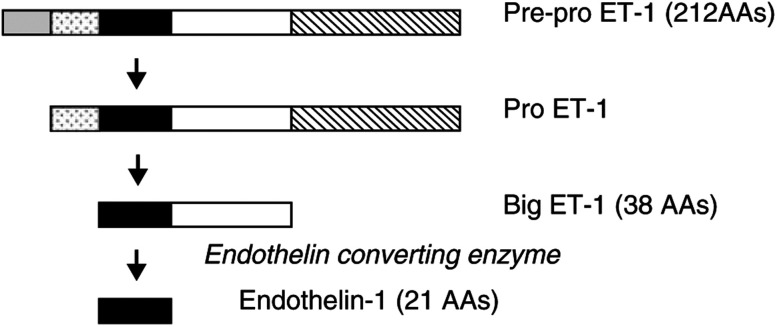
Biosynthesis of ET-1. The signal peptide (grey box) is cleaved from the amino terminus of the 212 amino-acid precursor molecule to generate pre-pro ET-1. Further cleavage, via furin-like endopeptidases including endothelin-converting enzyme (ECE), results in the generation of mature ET-1.

. The endothelins exert their physiological effect via two receptors, ET_A_ and ET_B_, which are G-protein-coupled transmembrane receptors found in both vascular and nonvascular tissues. Ligand-receptor binding induces dissociation of the receptor-linked G-protein subunits, which may then associate with multiple intracellular effectors. The ET_A_ receptor has varying affinities for the endothelin isoforms (ET-1>ET-2>ET-3), whereas the ET_B_ receptor shows no selective affinity for any of the ET subtypes (Sakamoto *et al*, 1991).

The endothelins have been implicated in numerous physiological and pathological conditions, including hypertension, cardiac failure and disseminated intravascular coagulation. Interest in the role of ET-1 in cancer has grown over the last decade, following the work of Kusuhara *et al* (1990) that demonstrated ET-1 production by several tumour cell lines. Currently there is evidence that ET-1 may modulate mitogenesis, apoptosis, angiogenesis, tumour invasion and development of metastases. The aim of this article is to review the role of ET-1 in cancer and possible ET-system modulation as an adjuvant therapeutic strategy.

## ENDOTHELIN EXPRESSION IN CANCER

Elevated plasma levels of ET-1 have been detected in patients with various solid tumours, including hepatocellular, gastric and prostate cancer (Nakamuta *et al*, 1993; Nelson *et al*, 1995, Ferrari-Bravo *et al*, 2000), where levels are greatest in patients with metastastic, hormone refractory disease.

Our group has demonstrated elevated plasma levels of ET-1 in patients with primary colorectal cancer, with and without liver metastases (Shankar *et al*, 1998), compared with healthy controls. Plasma levels of Big ET-1 have also been found to be significantly raised in colorectal cancer patients compared with age- and sex-matched controls (Simpson *et al*, 2000). Of note, this study also found that both preoperative and intraoperative portal plasma levels were significantly higher in Dukes' D carcinomas compared with localised or regional disease.

Many human cancer cell lines have been shown to synthesise ET-1 *in vitro*, including colonic, breast, stomach, prostate and glioblastoma cells (Kusuhara *et al*, 1990; Ali *et al*, 2000b). This is also reflected *in vivo*, where increased tissue immunoreactivity for ET-1 has been demonstrated in numerous cancer types, including ovarian, hepatocellular and breast tumours (Bagnato *et al*, 1999; Yamashita *et al*, 1991; Suzuki *et al*, 1998). We have reported increased immunopositivity for ET-1 in colorectal cancer sections. Of note, no correlation was noted between intensity of staining and Dukes' staging (Asham *et al*, 2001).

Furthermore, changes in the expression of endothelin system components have also been demonstrated in premalignant tissues. Egidy *et al* (2000) demonstrated, using reverse transcriptase polymerase chain reaction (RT–PCR), increased expression of pre-pro ET-1 and ECE mRNA in colorectal adenomas compared with normal colon. Also, Alanen *et al* (2000) demonstrated that immunoreactivity for ET-1 in breast ductal carcinoma *in situ* (DCIS) specimens was significantly higher (*P*<0.005) than that in normal breast tissue. A further significant increase in immunoreactivity was found in invasive tumours compared with DCIS (*P*<0.02). These results suggest that modulation of the endothelin system may be an early phenomenon in tumorigenesis.

## ENDOTHELIN RECEPTORS AND CANCER

Increased ET_A_ receptor expression in malignant tissue has been demonstrated using immunohistochemistry and/or autoradiography in several cancer types including colorectal, ovarian and prostate tumours (Nelson *et al*, 1996; Bagnato *et al*, 1999; Ali *et al*, 2000a). In the latter, levels of receptor expression have been found to correlate with both Gleason score and presence of metastases (Gohji *et al*, 2001).

Of note, in normal tissue from these sites the ET_B_ receptor predominates, whereas the ET_A_ receptor becomes prevalent in both primary tumours and metastases. Interestingly, relative hypermethylation of the ET_B_ gene has been demonstrated in several prostate, bladder and colon cancer cell lines. Furthermore, this has also been found to correlate with transcriptional downregulation (Pao *et al*, 2001), providing a plausible mechanism for reduced ET_B_ receptor expression in malignant tissue.

## ENDOTHELIN AS A MITOGEN

ET-1 has been shown to stimulate the growth of several human cancer cell lines *in vitro* including colorectal, ovarian, prostate, Kaposi's sarcoma and melanoma cells (Yohn *et al*, 1994; Nelson *et al*, 1996; Bagnato *et al*, 1999; Ali *et al*, 2000b; Bagnato *et al*, 2001). Several groups have demonstrated that in epithelial tumours *in vitro*, this mitogenic effect is mediated via the ET_A_ receptor (Nelson *et al*, 1996; Bagnato *et al*, 1999; Ali *et al*, 2000b).

The growth of nonepithelial tumours does not appear to be ET_A_ dependent. Studies on human melanoma cells have shown that the mitogenic effect of ET-1 is purely ET_B_ receptor dependent (Kikuchi *et al*, 1996), whereas antagonism of either receptor partially inhibits *in vitro* growth of Kaposi's sarcoma cells (Bagnato *et al*, 2001). This has also been demonstrated *in vivo*, where the specific ET_B_ antagonist (BQ788) was shown to significantly slow melanoma tumour growth in nude mice (Lahav *et al*, 1999).

**Figure 2:**
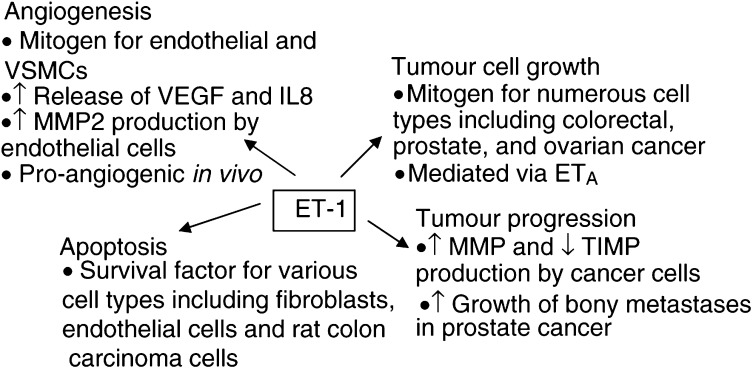
Actions of endothelin-1 in cancer.

The role of ET-1 as an autocrine growth factor has been demonstrated in human ovarian and colon cancer cell lines (Bagnato *et al*, 1995; Ali *et al*, 2000a). Furthermore, Moraitis *et al* (1999) have implicated ET-1 as a paracrine growth factor in ovarian cancer. They demonstrated that ET-1 production by human ovarian cancer cells stimulated the growth of carcinoma-associated fibroblasts in coculture, an effect that was partially inhibited by both ET_A_ and ET_B_ antagonism. However, a recent study by Kernochan *et al* (2002) found that ET-1 has no effect on human colonic subepithelial myofibroblast proliferation, although contraction and migration of these cells was stimulated through ET receptor-mediated myosin phosphorylation. The effects of ET-1 on proliferation and other cellular processes in cancer are summarised in Figure 2

.

## ENDOTHELIN AND APOPTOSIS

In addition to its mitogenic effect, there is evidence that ET-1 may also contribute to tumour growth by protecting cells from apoptosis. ET-1 has been shown to protect rat fibroblasts and human endothelial cells (Wu-Wong *et al*, 1997) from serum-deprivation-induced apoptosis *in vitro* (Shichiri *et al*, 1997). Peduto-Eberl *et al* (2000) have also more recently demonstrated that ET-1 is a survival factor for rat colon carcinoma cells against FasL-mediated apoptosis. From these data, it could be suggested that ET-1 may influence tumour growth by influencing both cellular proliferation and cell death.

## ENDOTHELIN AND ANGIOGENESIS

Endothelin-1 may also facilitate tumour growth through the promotion of angiogenesis. ET-1 is a potent mitogen for both endothelial cells and vascular smooth muscle cells (VSMC) *in vitro* (Komuro *et al*, 1988; Pedram *et al*, 1997). In addition, ET-1 may indirectly enhance endothelial cell proliferation through stimulation of vascular endothelial growth factor (VEGF) production by other cell types (Pedram *et al*, 1997; Salani *et al*, 2000a). The reverse situation has also been demonstrated in endothelial cells, where VEGF has been shown to enhance ET-1 mRNA expression and ET-1 secretion (Matsuura *et al*, 1998).

Furthermore, ET-1 potentiates the effect of several proangiogenic factors *in vitro*, including PDGF and VEGF (Pedram *et al*, 1997; Yang *et al*, 1999). ET-1 also stimulates invasion and morphological differentiation of human umbilical vein endothelial cells (HUVEC) in matrigel *in vitro*, and this may be facilitated via ET-1-induced production of matrix metalloproteinase-2 (MMP-2) by endothelial cells (Salani *et al*, 2000b).

*In vivo*, when combined with VEGF, ET-1 has been shown to stimulate angiogenesis in subcutaneously implanted matrigel plugs in mice (Salani *et al*, 2000b). Bek and McMillen (2000) demonstrated that ET-1 also stimulated angiogenesis in a rat corneal model with a similar efficacy to VEGF. In this model they found that ET-1-stimulated angiogenesis was inhibited by either ET_A_ antagonism, or mixed antagonism with bosentan, but was not affected by the addition of an ET_B_ antagonist. These data suggest that ET-1 may be an important modulator of angiogenesis in cancer.

## ENDOTHELIN-1 AND TUMOUR PROGRESSION/METASTASES

There is increasing evidence that ET-1 may also influence tumour invasion and metastases. A recent study in human ovarian carcinoma cell lines has demonstrated that ET-1 can regulate the expression of several MMPs, in particular, MMP-2 and MMP-9, and can downregulate tissue inhibitors of matrix metalloproteinases (TIMP) 1 and 2 (Rosano *et al*, 2001).

ET-1 may also modulate the growth of bony metastases from prostate cancer. In human prostate cancer cells, ET-1 production is enhanced by bone contact, which in turn blocks osteoclastic bone reabsorption (Chiao *et al*, 2000). This is also reflected *in vivo*, where Nelson *et al* (1999) used an osteoblastic tumour model (WISH–a human tumour derived from amnion) to demonstrate that tumours transfected to overexpress ET-1 produced significantly more bone growth in nude mice compared with vector only controls.

Furthermore, our group has demonstrated increased immunoreactivity for ET-1 in endothelial cells within colorectal liver metastases compared with surrounding vessels (Shankar *et al*, 1998), suggesting that ET-1 may be involved in modulation of tumour blood flow, known to be altered in liver metastases.

## ENDOTHELIN ANTAGONISM AS A THERAPEUTIC STRATEGY

Several *in vivo* models have been used to assess the role of endothelin antagonism in tumorigenesis. Work originating from our department using intraportally injected syngeneic MC28 cells in rats demonstrated that ET_A_ antagonism with BQ123 significantly reduced hepatic tumour load compared with controls (Asham *et al*, 2001).

Peduto-Eberl *et al* (2000) assessed the effect of bosentan, a dual receptor antagonist, on the growth of peritoneal tumours derived from a syngeneic rat colonic adenocarcinoma cell line. Although bosentan was not able to control tumour progression, they did find that tumours were generally of lower grade, and there were fewer spontaneous deaths in the treated *vs* the untreated groups. Egidy *et al* (2000) used the same tumour model to assess histological differences between tumours of bosentan-treated animals and controls. They demonstrated that tumour cells were less densely packed, and there was less collagen matrix around tumour nodules in the treated compared to the untreated group.

Finally, using an osteoblastic tumour model in nude mice Nelson *et al* (1999) have shown that ET_A_ antagonism with A127722 significantly reduced the growth of new bone compared with vehicle treated controls. Although *in vivo* results have so far not yielded dramatic results, they are encouraging and warrant further investigation.

Recently, a phase I trial of the ET_A_ receptor antagonist atrasentan was undertaken in 31 patients with refractory adenocarcinomas (Carducci *et al*, 2002). Nearly half of the patients had prostate cancer (*n*=14), although patients with other malignancies, including colorectal (*n*=6), breast (*n*=2), lung (*n*=4) and renal cell carcinoma (*n*=3), were recruited. Side effects relating to the physiological consequences of ET_A_ blockade include headache, hypotension and peripheral oedema that were generally tolerated, being mild to moderate in nature. Of the 24 patients who completed the initial 28-day trial, no complete or partial radiological responses were observed. However, a third of patients with tumour-related pain experienced alleviation of symptoms. Additionally, prostatic specific antigen (PSA) levels were found to fall in half of the prostate cancer patients, and reduction in other biochemical tumour markers such as CEA and CA125 were also recorded, suggesting antitumour activity. It remains to be seen whether this will result in a significant clinical benefit.

## CONCLUSION

Components of the endothelin system are altered in cancer, and appear to aid tumour growth and progression in a number of epithelial cancer types, via direct and indirect mechanisms. From the evidence to date, it appears that selective ET_A_ antagonism provides the most likely effective method of endothelin system inhibition in cancer. With generally mild to moderate side effects, and suggested antitumour activity, further development and clinical evaluation of these agents is warranted to determine possible therapeutic potential as an adjuvant anticancer strategy.
